# 2-(7-Methyl-3-oxo-1-phenyl­perhydro­naphthalen-4a-yl)malononitrile

**DOI:** 10.1107/S1600536809047175

**Published:** 2009-11-11

**Authors:** Tai-Ran Kang, Lian-Mei Chen

**Affiliations:** a College of Chemistry and Chemical Engineering, China West Normal University, Nanchong 637002, People’s Republic of China

## Abstract

In the title compound, C_20_H_22_N_2_O, both cyclo­hexane rings adopt chair conformations. Weak C—H⋯N and C—H⋯O hydrogen bonding is present in the crystal structure.

## Related literature

For the use of malononitrile-containing compounds as building blocks in organic synthesis, see: Magdi *et al.* (2003 ▶); Michail & Sergey (2008 ▶); Zhang *et al.* (2008 ▶). For a related structure, see: Zhou *et al.* (2007 ▶).
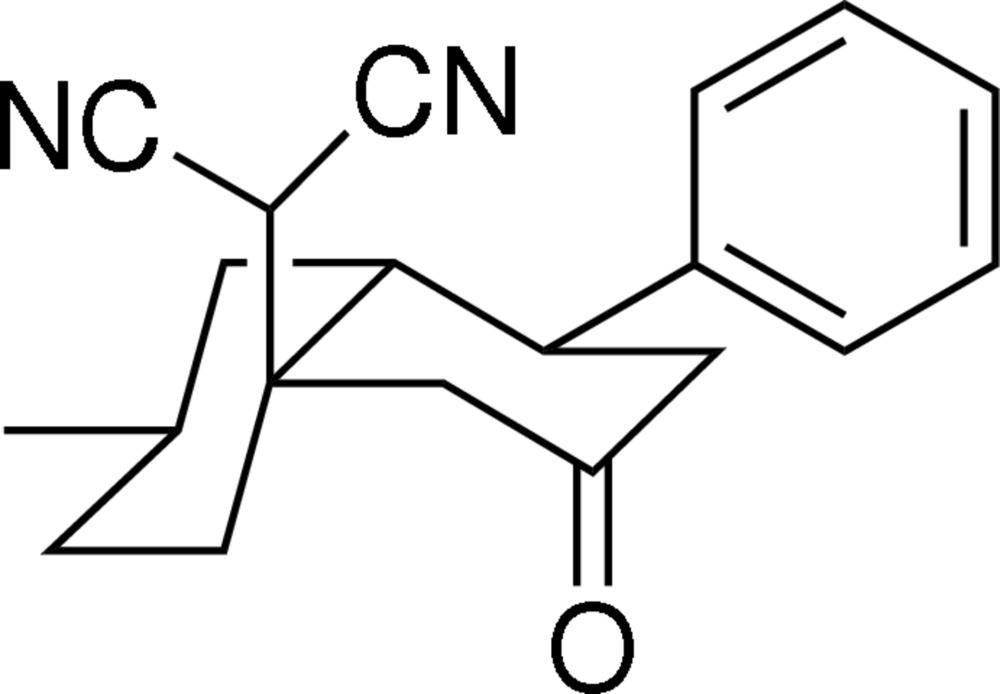


## Experimental

### 

#### Crystal data

C_20_H_22_N_2_O
*M* *_r_* = 306.40
Monoclinic, 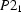

*a* = 11.575 (2) Å
*b* = 6.0907 (12) Å
*c* = 12.276 (3) Å
β = 101.38 (3)°
*V* = 848.4 (3) Å^3^
*Z* = 2
Mo *K*α radiation
μ = 0.07 mm^−1^
*T* = 113 K
0.25 × 0.24 × 0.21 mm

#### Data collection

Rigaku Saturn CCD area-detector diffractometer
Absorption correction: none
7044 measured reflections
2194 independent reflections
1479 reflections with *I* > 2σ(*I*)
*R*_int_ = 0.063

#### Refinement

*R*[*F* ^2^ > 2σ(*F* ^2^)] = 0.039
*wR*(*F* ^2^) = 0.083
*S* = 1.04
2194 reflections
209 parameters
1 restraint
H-atom parameters constrained
Δρ_max_ = 0.22 e Å^−3^
Δρ_min_ = −0.24 e Å^−3^

### 

Data collection: *CrystalClear* (Rigaku/MSC, 2005 ▶); cell refinement: *CrystalClear*; data reduction: *CrystalClear*; program(s) used to solve structure: *SHELXS97* (Sheldrick, 2008 ▶); program(s) used to refine structure: *SHELXL97* (Sheldrick, 2008 ▶); molecular graphics: *ORTEP-3* (Farrugia, 1997 ▶); software used to prepare material for publication: *SHELXL97*.

## Supplementary Material

Crystal structure: contains datablocks global, I. DOI: 10.1107/S1600536809047175/xu2670sup1.cif

Structure factors: contains datablocks I. DOI: 10.1107/S1600536809047175/xu2670Isup2.hkl

Additional supplementary materials:  crystallographic information; 3D view; checkCIF report

## Figures and Tables

**Table 1:** Hydrogen-bond geometry (Å, °)

*D*—H⋯*A*	*D*—H	H⋯*A*	*D*⋯*A*	*D*—H⋯*A*
C9—H9⋯O1^i^	0.95	2.54	3.451 (3)	162
C12—H12*A*⋯O1^ii^	0.99	2.35	3.159 (2)	138
C18—H18⋯N1^iii^	1.00	2.36	3.306 (3)	157

Symmetry codes: (i) 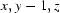; (ii) 
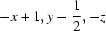
; (iii) 
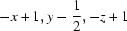
.

**Fig. 1.**
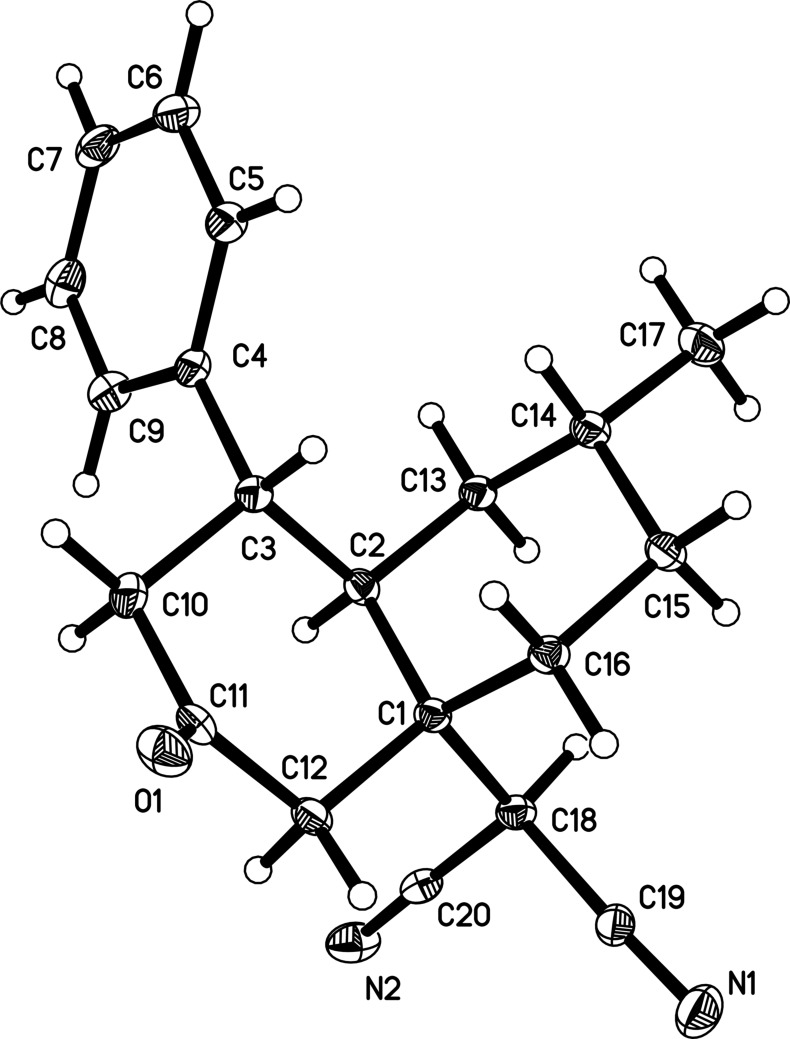
The molecular structure of (I) with 30% probability displacement ellipsoids (arbitrary spheres for H atoms).

## supplementary crystallographic information

### Comment

Malononitrile derivatives are useful intermediates in organic synthesis (Michail *et al.* 2008; Zhang *et al.* 2008; Zhou *et al.* 2007). Their potential applications are used for the preparation of heterocyclic ring compounds (Magdi *et al.* 2003). As a part of our interest in the synthsis of some complex ring systems, we investigated the title compound, (I), which is a potential precursor in the preparation of multifunctional tricyclic compound. We report herein the crystal structure of the title compound.

The molecular structure of (I) is shown in Fig. 1. Bond lengths and angles in (I) are normal. Two six membered rings (cyclohexanone and cyclohexane) adopt an chair conformation. The crystal packing is stabilized by C—H···N and C—H···0 hydrogen bonding (Table 1).

### Experimental

2-(4-methylcyclohexylidene)malononitrile (0.16 g, 1 mmol), (*E*)-4-phenylbut-3-en-2-one (0.175 g, 1.2 mmol), 9S-amino-9-deoxyepiquinine (0.065 g, 0.2 mmol), 2,2,2-trifluoroacetic acid (0.029 g, 0.4 mmol) and *N*-ethyl-*N*-isopropylpropan-2-amine (0.023 g, 0.15 mmol) were stirred in THF (3 ml) at 298 K for 110 h. Then the reaction was quenched by adding 1 mol/*L* HCl (5 ml). The mixture was extracted with ethyl acetate (20 ml), dried with anhydrous sodium sulfate. The solvent was removed under reduced pressure and flash chromatography on silica gel gave the pure compound as a white solid. Colorless single crystals of (I) suitable for X-ray analysis were obtained by slow evaporation the mixture solvents of ethyl acetate and petroleum ether.

### Refinement

The carbon-bound hydrogen atoms were placed in calculated positions, with C—H = 0.95–1.00 Å, and refined using a riding model, with *U*_iso_(H) =1.5*U*_eq_(C) for methyl H atom and 1.2*U*_eq_(C) for the others. In the absence of significant anomalous scattering effects, Friedel pairs were averaged.

### Crystal data

**Table:**

C_20_H_22_N_2_O	*F*(000) = 328
*M**_r_* = 306.40	*D*_x_ = 1.199 Mg m^−^^3^
Monoclinic, *P*2_1_	Mo *K*α radiation, λ = 0.71073 Å
Hall symbol: P 2yb	Cell parameters from 2724 reflections
*a* = 11.575 (2) Å	θ = 3.4–27.9°
*b* = 6.0907 (12) Å	µ = 0.07 mm^−^^1^
*c* = 12.276 (3) Å	*T* = 113 K
β = 101.38 (3)°	Block, colourless
*V* = 848.4 (3) Å^3^	0.25 × 0.24 × 0.21 mm
*Z* = 2	

### Data collection

**Table:**

Rigaku Saturn CCD area-detector diffractometer	1479 reflections with *I* > 2σ(*I*)
Radiation source: rotating anode	*R*_int_ = 0.063
confocal	θ_max_ = 27.9°, θ_min_ = 3.4°
Detector resolution: 7.31 pixels mm^-1^	*h* = −15→15
ω and φ scans	*k* = −7→7
7044 measured reflections	*l* = −14→16
2194 independent reflections	

### Refinement

**Table:**

Refinement on *F*^2^	Primary atom site location: structure-invariant direct methods
Least-squares matrix: full	Secondary atom site location: difference Fourier map
*R*[*F*^2^ > 2σ(*F*^2^)] = 0.039	Hydrogen site location: inferred from neighbouring sites
*wR*(*F*^2^) = 0.083	H-atom parameters constrained
*S* = 1.04	*w* = 1/[σ^2^(*F*_o_^2^) + (0.021*P*)^2^] where *P* = (*F*_o_^2^ + 2*F*_c_^2^)/3
2194 reflections	(Δ/σ)_max_ < 0.001
209 parameters	Δρ_max_ = 0.22 e Å^−^^3^
1 restraint	Δρ_min_ = −0.24 e Å^−^^3^

### Special details

**Table:**

Geometry. All e.s.d.'s (except the e.s.d. in the dihedral angle between two l.s. planes) are estimated using the full covariance matrix. The cell e.s.d.'s are taken into account individually in the estimation of e.s.d.'s in distances, angles and torsion angles; correlations between e.s.d.'s in cell parameters are only used when they are defined by crystal symmetry. An approximate (isotropic) treatment of cell e.s.d.'s is used for estimating e.s.d.'s involving l.s. planes.
Refinement. Refinement of *F*^2^ against ALL reflections. The weighted *R*-factor *wR* and goodness of fit *S* are based on *F*^2^, conventional *R*-factors *R* are based on *F*, with *F* set to zero for negative *F*^2^. The threshold expression of *F*^2^ > σ(*F*^2^) is used only for calculating *R*-factors(gt) *etc*. and is not relevant to the choice of reflections for refinement. *R*-factors based on *F*^2^ are statistically about twice as large as those based on *F*, and *R*- factors based on ALL data will be even larger.

### Fractional atomic coordinates and isotropic or equivalent isotropic displacement parameters (Å^2^)

**Table:**

	*x*	*y*	*z*	*U*_iso_*/*U*_eq_	
O1	0.59916 (14)	0.6258 (3)	0.05489 (13)	0.0319 (4)	
N1	0.3799 (2)	0.3869 (4)	0.41898 (18)	0.0389 (6)	
N2	0.40092 (18)	−0.1712 (4)	0.21854 (19)	0.0354 (6)	
C1	0.61036 (19)	0.2632 (4)	0.27728 (18)	0.0183 (5)	
C2	0.71015 (19)	0.1075 (4)	0.25731 (17)	0.0193 (5)	
H2	0.6728	−0.0267	0.2185	0.023*	
C3	0.7857 (2)	0.2188 (4)	0.18092 (18)	0.0204 (5)	
H3	0.8198	0.3556	0.2194	0.025*	
C4	0.8884 (2)	0.0760 (4)	0.16412 (18)	0.0223 (6)	
C5	1.0035 (2)	0.1440 (4)	0.20656 (19)	0.0272 (6)	
H5	1.0169	0.2812	0.2437	0.033*	
C6	1.0987 (2)	0.0139 (5)	0.1952 (2)	0.0330 (7)	
H6	1.1766	0.0634	0.2239	0.040*	
C7	1.0812 (2)	−0.1863 (5)	0.1427 (2)	0.0329 (7)	
H7	1.1466	−0.2765	0.1367	0.040*	
C8	0.9677 (2)	−0.2551 (4)	0.0988 (2)	0.0330 (6)	
H8	0.9550	−0.3919	0.0612	0.040*	
C9	0.8720 (2)	−0.1242 (4)	0.10967 (19)	0.0283 (6)	
H9	0.7943	−0.1729	0.0793	0.034*	
C10	0.7070 (2)	0.2892 (4)	0.07005 (18)	0.0261 (6)	
H10A	0.7555	0.3644	0.0234	0.031*	
H10B	0.6711	0.1578	0.0294	0.031*	
C11	0.6119 (2)	0.4407 (4)	0.09118 (19)	0.0239 (6)	
C12	0.5360 (2)	0.3476 (4)	0.16652 (17)	0.0220 (5)	
H12A	0.4884	0.2251	0.1281	0.026*	
H12B	0.4811	0.4625	0.1824	0.026*	
C13	0.7868 (2)	0.0355 (4)	0.36900 (18)	0.0207 (5)	
H13A	0.7386	−0.0562	0.4094	0.025*	
H13B	0.8525	−0.0563	0.3540	0.025*	
C14	0.8377 (2)	0.2275 (4)	0.44301 (19)	0.0233 (6)	
H14	0.8921	0.3100	0.4039	0.028*	
C15	0.7405 (2)	0.3855 (4)	0.46116 (18)	0.0226 (5)	
H15A	0.7768	0.5170	0.5013	0.027*	
H15B	0.6912	0.3132	0.5080	0.027*	
C16	0.6622 (2)	0.4565 (4)	0.35109 (18)	0.0207 (5)	
H16A	0.7093	0.5488	0.3097	0.025*	
H16B	0.5969	0.5477	0.3673	0.025*	
C17	0.9091 (2)	0.1482 (5)	0.55366 (19)	0.0341 (7)	
H17A	0.8580	0.0640	0.5932	0.051*	
H17B	0.9414	0.2750	0.5987	0.051*	
H17C	0.9737	0.0546	0.5403	0.051*	
C18	0.52557 (19)	0.1314 (4)	0.33947 (18)	0.0213 (5)	
H18	0.5755	0.0568	0.4048	0.026*	
C19	0.4415 (2)	0.2767 (4)	0.38201 (19)	0.0255 (6)	
C20	0.4563 (2)	−0.0388 (4)	0.2708 (2)	0.0235 (6)	

### Atomic displacement parameters (Å^2^)

**Table:**

	*U*^11^	*U*^22^	*U*^33^	*U*^12^	*U*^13^	*U*^23^
O1	0.0308 (10)	0.0322 (11)	0.0298 (9)	0.0017 (9)	−0.0009 (8)	0.0110 (9)
N1	0.0373 (14)	0.0422 (15)	0.0404 (13)	−0.0023 (12)	0.0155 (11)	−0.0142 (12)
N2	0.0259 (12)	0.0288 (14)	0.0513 (14)	−0.0010 (11)	0.0073 (11)	−0.0060 (12)
C1	0.0163 (12)	0.0199 (13)	0.0181 (11)	0.0011 (10)	0.0022 (9)	0.0019 (10)
C2	0.0195 (12)	0.0195 (13)	0.0180 (11)	−0.0005 (11)	0.0010 (9)	−0.0021 (11)
C3	0.0183 (12)	0.0233 (14)	0.0196 (11)	0.0020 (11)	0.0035 (10)	0.0007 (11)
C4	0.0211 (13)	0.0274 (16)	0.0196 (12)	−0.0006 (12)	0.0070 (10)	0.0021 (11)
C5	0.0239 (13)	0.0336 (15)	0.0247 (12)	−0.0001 (13)	0.0063 (10)	0.0019 (12)
C6	0.0208 (14)	0.0470 (19)	0.0315 (14)	0.0015 (13)	0.0059 (11)	0.0048 (13)
C7	0.0273 (15)	0.0419 (19)	0.0326 (14)	0.0129 (14)	0.0133 (12)	0.0085 (13)
C8	0.0396 (17)	0.0318 (15)	0.0309 (14)	0.0053 (14)	0.0150 (13)	−0.0003 (13)
C9	0.0258 (14)	0.0293 (15)	0.0309 (14)	0.0007 (12)	0.0086 (11)	−0.0004 (12)
C10	0.0274 (14)	0.0332 (15)	0.0192 (12)	0.0010 (12)	0.0080 (10)	0.0044 (11)
C11	0.0213 (13)	0.0314 (16)	0.0164 (11)	0.0014 (12)	−0.0030 (10)	0.0017 (11)
C12	0.0190 (12)	0.0245 (14)	0.0200 (12)	0.0018 (12)	−0.0019 (10)	−0.0005 (11)
C13	0.0181 (12)	0.0232 (14)	0.0204 (12)	0.0028 (11)	0.0030 (10)	0.0020 (10)
C14	0.0193 (12)	0.0283 (14)	0.0211 (12)	0.0004 (11)	0.0012 (10)	0.0020 (11)
C15	0.0204 (13)	0.0235 (14)	0.0224 (12)	0.0002 (11)	0.0001 (10)	−0.0021 (11)
C16	0.0190 (12)	0.0195 (13)	0.0232 (12)	0.0018 (11)	0.0032 (10)	−0.0012 (11)
C17	0.0278 (14)	0.0434 (17)	0.0275 (13)	0.0072 (14)	−0.0036 (11)	−0.0005 (13)
C18	0.0178 (11)	0.0218 (12)	0.0239 (12)	0.0023 (11)	0.0031 (10)	0.0000 (11)
C19	0.0228 (13)	0.0311 (15)	0.0237 (12)	−0.0063 (13)	0.0070 (11)	−0.0056 (12)
C20	0.0192 (13)	0.0230 (14)	0.0295 (14)	0.0035 (12)	0.0081 (11)	0.0008 (12)

### Geometric parameters (Å, °)

**Table:**

O1—C11	1.211 (3)	C10—C11	1.498 (3)
N1—C19	1.137 (3)	C10—H10A	0.9900
N2—C20	1.145 (3)	C10—H10B	0.9900
C1—C16	1.534 (3)	C11—C12	1.506 (3)
C1—C12	1.547 (3)	C12—H12A	0.9900
C1—C2	1.551 (3)	C12—H12B	0.9900
C1—C18	1.577 (3)	C13—C14	1.526 (3)
C2—C13	1.543 (3)	C13—H13A	0.9900
C2—C3	1.558 (3)	C13—H13B	0.9900
C2—H2	1.0000	C14—C17	1.522 (3)
C3—C4	1.520 (3)	C14—C15	1.530 (3)
C3—C10	1.542 (3)	C14—H14	1.0000
C3—H3	1.0000	C15—C16	1.533 (3)
C4—C9	1.386 (3)	C15—H15A	0.9900
C4—C5	1.395 (3)	C15—H15B	0.9900
C5—C6	1.386 (3)	C16—H16A	0.9900
C5—H5	0.9500	C16—H16B	0.9900
C6—C7	1.375 (4)	C17—H17A	0.9800
C6—H6	0.9500	C17—H17B	0.9800
C7—C8	1.384 (4)	C17—H17C	0.9800
C7—H7	0.9500	C18—C20	1.470 (3)
C8—C9	1.392 (3)	C18—C19	1.484 (3)
C8—H8	0.9500	C18—H18	1.0000
C9—H9	0.9500		
			
C16—C1—C12	110.45 (19)	C11—C12—C1	111.99 (18)
C16—C1—C2	110.22 (18)	C11—C12—H12A	109.2
C12—C1—C2	111.52 (17)	C1—C12—H12A	109.2
C16—C1—C18	108.26 (17)	C11—C12—H12B	109.2
C12—C1—C18	107.66 (17)	C1—C12—H12B	109.2
C2—C1—C18	108.61 (18)	H12A—C12—H12B	107.9
C13—C2—C1	110.43 (16)	C14—C13—C2	113.48 (19)
C13—C2—C3	111.48 (18)	C14—C13—H13A	108.9
C1—C2—C3	110.66 (18)	C2—C13—H13A	108.9
C13—C2—H2	108.1	C14—C13—H13B	108.9
C1—C2—H2	108.1	C2—C13—H13B	108.9
C3—C2—H2	108.1	H13A—C13—H13B	107.7
C4—C3—C10	112.40 (17)	C17—C14—C13	111.5 (2)
C4—C3—C2	112.31 (18)	C17—C14—C15	110.78 (19)
C10—C3—C2	110.42 (18)	C13—C14—C15	111.13 (19)
C4—C3—H3	107.1	C17—C14—H14	107.8
C10—C3—H3	107.1	C13—C14—H14	107.8
C2—C3—H3	107.1	C15—C14—H14	107.8
C9—C4—C5	118.1 (2)	C14—C15—C16	111.88 (18)
C9—C4—C3	122.2 (2)	C14—C15—H15A	109.2
C5—C4—C3	119.7 (2)	C16—C15—H15A	109.2
C6—C5—C4	120.8 (2)	C14—C15—H15B	109.2
C6—C5—H5	119.6	C16—C15—H15B	109.2
C4—C5—H5	119.6	H15A—C15—H15B	107.9
C7—C6—C5	120.5 (2)	C15—C16—C1	113.49 (18)
C7—C6—H6	119.7	C15—C16—H16A	108.9
C5—C6—H6	119.7	C1—C16—H16A	108.9
C6—C7—C8	119.4 (2)	C15—C16—H16B	108.9
C6—C7—H7	120.3	C1—C16—H16B	108.9
C8—C7—H7	120.3	H16A—C16—H16B	107.7
C7—C8—C9	120.1 (3)	C14—C17—H17A	109.5
C7—C8—H8	119.9	C14—C17—H17B	109.5
C9—C8—H8	119.9	H17A—C17—H17B	109.5
C4—C9—C8	120.9 (2)	C14—C17—H17C	109.5
C4—C9—H9	119.5	H17A—C17—H17C	109.5
C8—C9—H9	119.5	H17B—C17—H17C	109.5
C11—C10—C3	110.17 (18)	C20—C18—C19	107.53 (19)
C11—C10—H10A	109.6	C20—C18—C1	113.71 (18)
C3—C10—H10A	109.6	C19—C18—C1	112.4 (2)
C11—C10—H10B	109.6	C20—C18—H18	107.7
C3—C10—H10B	109.6	C19—C18—H18	107.7
H10A—C10—H10B	108.1	C1—C18—H18	107.7
O1—C11—C10	123.4 (2)	N1—C19—C18	177.1 (3)
O1—C11—C12	122.2 (2)	N2—C20—C18	178.7 (2)
C10—C11—C12	114.3 (2)		
			
C16—C1—C2—C13	54.0 (2)	C3—C10—C11—O1	120.9 (2)
C12—C1—C2—C13	177.03 (19)	C3—C10—C11—C12	−56.3 (3)
C18—C1—C2—C13	−64.5 (2)	O1—C11—C12—C1	−124.1 (2)
C16—C1—C2—C3	−69.9 (2)	C10—C11—C12—C1	53.1 (3)
C12—C1—C2—C3	53.1 (2)	C16—C1—C12—C11	72.3 (2)
C18—C1—C2—C3	171.61 (17)	C2—C1—C12—C11	−50.7 (3)
C13—C2—C3—C4	53.7 (2)	C18—C1—C12—C11	−169.7 (2)
C1—C2—C3—C4	177.04 (18)	C1—C2—C13—C14	−55.3 (2)
C13—C2—C3—C10	−179.95 (19)	C3—C2—C13—C14	68.2 (2)
C1—C2—C3—C10	−56.6 (2)	C2—C13—C14—C17	178.06 (19)
C10—C3—C4—C9	−60.6 (3)	C2—C13—C14—C15	53.9 (2)
C2—C3—C4—C9	64.7 (3)	C17—C14—C15—C16	−176.4 (2)
C10—C3—C4—C5	120.9 (2)	C13—C14—C15—C16	−51.9 (3)
C2—C3—C4—C5	−113.8 (2)	C14—C15—C16—C1	53.7 (3)
C9—C4—C5—C6	−0.5 (3)	C12—C1—C16—C15	−178.17 (18)
C3—C4—C5—C6	178.1 (2)	C2—C1—C16—C15	−54.5 (2)
C4—C5—C6—C7	−0.7 (4)	C18—C1—C16—C15	64.2 (2)
C5—C6—C7—C8	1.5 (4)	C16—C1—C18—C20	171.35 (19)
C6—C7—C8—C9	−1.2 (4)	C12—C1—C18—C20	51.9 (2)
C5—C4—C9—C8	0.8 (3)	C2—C1—C18—C20	−69.0 (2)
C3—C4—C9—C8	−177.8 (2)	C16—C1—C18—C19	48.9 (2)
C7—C8—C9—C4	0.1 (4)	C12—C1—C18—C19	−70.5 (2)
C4—C3—C10—C11	−176.4 (2)	C2—C1—C18—C19	168.59 (18)
C2—C3—C10—C11	57.3 (3)		

### Hydrogen-bond geometry (Å, °)

**Table:**

*D*—H···*A*	*D*—H	H···*A*	*D*···*A*	*D*—H···*A*
C9—H9···O1^i^	0.95	2.54	3.451 (3)	162
C12—H12A···O1^ii^	0.99	2.35	3.159 (2)	138
C18—H18···N1^iii^	1.00	2.36	3.306 (3)	157

Symmetry codes: (i) *x*, *y*−1, *z*; (ii) −*x*+1, *y*−1/2, −*z*; (iii) −*x*+1, *y*−1/2, −*z*+1.
